# Retrosplenial cortex and aversive conditioning

**DOI:** 10.3389/fnbeh.2024.1341705

**Published:** 2024-06-25

**Authors:** Han Yin Cheng, Danielle I. Fournier, Travis P. Todd

**Affiliations:** Department of Psychological Science, University of Vermont, Burlington, VT, United States

**Keywords:** retrosplenial cortex, avoidance, Pavlovian fear conditioning, aversive conditioning, context

## Abstract

The retrosplenial cortex (RSC) is well-known for its contribution to episodic memory, as well as contextual and spatial learning and memory. However, two literatures have also emerged examining the role of the RSC in aversive conditioning. The purpose of this manuscript is to review, and attempt to integrate, these two literatures. We focus on studies in which discrete cues, such as tones, predict the occurrence of aversive outcomes, such as mild shocks. Using both electrophysiological recordings and lesion methods, the first literature has examined RSC contributions to discriminative avoidance conditioning. The second, and more recent literature, has focused on the role of the RSC in Pavlovian fear conditioning. We discuss both literatures in terms of the type of information processed by the RSC, the role of the RSC in memory storage, and how the aversive conditioning literature might be consistent with a role for the RSC in contextual learning and memory.

## Introduction

Aversive conditioning, or learning about dangerous or harmful outcomes, is a critical foundation for organizing adaptive behavior and can be established via Pavlovian or instrumental learning processes. For instance, through Pavlovian conditioning, organisms can learn the predictive relationship between stimuli in the environment and aversive outcomes, and thus organize behavior to prepare for these outcomes (Fanselow et al., 2019). In addition, through instrumental conditioning, organisms can learn about the consequences of their own actions. In this case, they can avoid performing future actions that produce dangerous or aversive outcomes (Cain, 2019).

There has been considerable research aimed at understanding the neural circuits that control aversive conditioning. As noted, Pavlovian conditioning is one way in which organisms learn to predict the occurrence of dangerous outcomes and is perhaps the most well-studied form of aversive conditioning. In studies with rodents, a standard procedure involves pairings of an auditory stimulus, such as a tone, with a mild footshock. This typically results in the tone acquiring conditioned stimulus (CS) properties, with the ability to elicit defensive behaviors. Auditory fear conditioning has been extensively studied and is supported by a circuit that includes the thalamus, amygdala, and brainstem (Bouton et al., 2021).

In addition to these regions, additional research has focused on frontal cortical and hippocampal contributions to aversive learning and memory (Maren et al., 2013). However, less is known about the role of more posterior cortical regions, including the retrosplenial cortex (RSC). While the RSC is perhaps best known for its role in spatial navigation (Vann et al., 2009), it has become clear that RSC function extends beyond navigation to include other cognitive functions such as episodic memory (see Alexander et al., 2023). Furthermore, recent studies suggest the RSC makes critical contributions to some forms of aversive conditioning (e.g., Todd et al., 2016; Trask and Helmstetter, 2022).

The purpose of the present paper is to therefore review and integrate research that has examined RSC contributions to aversive conditioning. An extensive literature has focused on the RSC and *contextual* fear conditioning, which will not be reviewed here (for a review see Corcoran et al., 2018; see also Trask and Helmstetter, 2022). Instead, this review will focus on studies of aversive conditioning to *discrete* cues, such as auditory or visual stimuli. The first section of this paper will review and summarize findings by M. Gabriel and his colleagues, who in an extensive collection of studies examined the neural circuitry of discriminative avoidance learning in rabbits (see Gabriel, 1993). In this section we provide a detailed narrative of these studies, in part due to the scope of the experiments, but also to highlight what we view are key findings. The second section of this paper reports on the main findings from recent experiments that have extended this prior research by examining the role of the RSC in Pavlovian fear conditioning. We end with an attempt to integrate these two lines of research, by discussing potential roles of the RSC in aversive conditioning.

## Part 1: RSC activity and contributions to discriminative avoidance learning

### Nomenclature clarification

**Figure 1:**
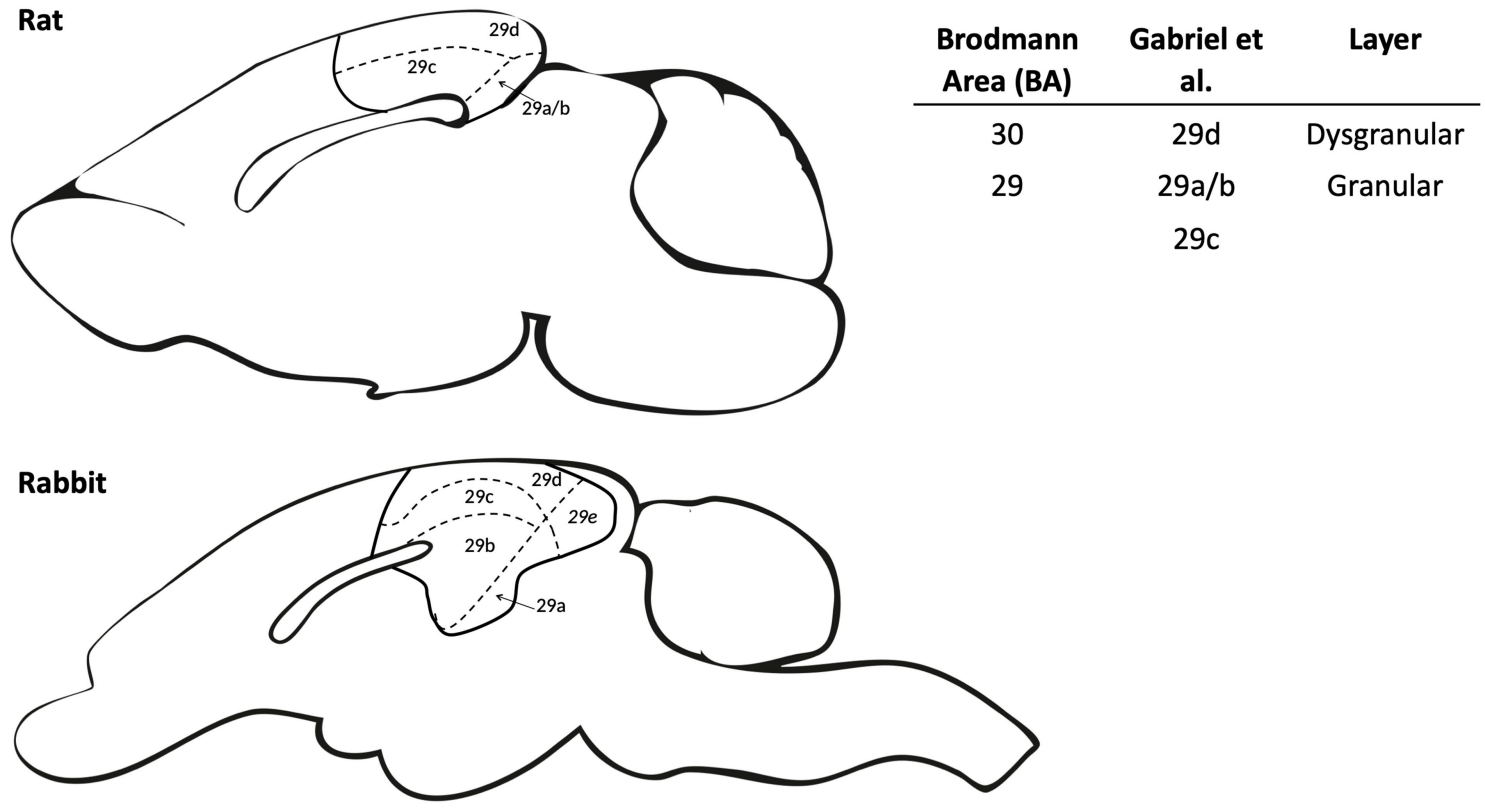
Schematic comparing retrosplenial cortex (RSC) subdivisions between rat and rabbit. The RSC anatomy is divided into dysgranular and granular subregions for both rat and rabbit. The dysgranular region (BA 30) was identified by Gabriel and colleagues as area 29d, which has a pronounced disorganized and underdeveloped layer IV in the rat and medial IV in rabbits (Vogt et al., 1986). Granular regions (BA 29) were identified by Gabriel and colleagues as areas 29a/b/c which are largely homologous between the rat and rabbit. Area 29e in the rabbit does not have an equivalent in the rat and was not a target for neural recording in the relevant literature. The right panel compares RSC nomenclature for the various subdivisions among commonly cited literature.

In studies with rabbits, Gabriel and colleagues often used the term “posterior cingulate” to refer to Brodmann area 29, although the term “retrosplenial cortex” is more commonly used in recent rodent literature (Smith et al. 2018). Gabriel’s work typically involved recording in rabbit areas 29b/c/d, which refers to distinct subregions of the RSC that are defined morphologically. In particular, the RSC can be divided into two distinct subregions based on the presence or absence of a granular layer. In Gabriel’s work, area 29d referred to the dysgranular RSC (dRSC; Brodmann 30), which is located dorsally and lacks a distinct granular cell layer. Meanwhile, the granular RSC (gRSC) has been further divided into two or three subregions depending on the literature referenced, with the most dorsal (closest to dRSC) referred to as area 29c and the most ventral granular RSC subregions referred to as 29b (or with a smaller subregion known as area 29a in some literature; Figure 1; Vogt and Peters, 1981; Van Groen and Wyss, 2003; Malinowska et al., 2016). For this review, we will focus on describing functions of the RSC as a whole, though we will highlight sub-regions where differential processing/contributions may exist.

### Overview of behavioral procedures

As noted, Gabriel and colleagues conducted an extensive series of studies examining the neural circuits controlling discriminative avoidance learning. In this procedure, rabbits were placed in a running wheel, and received presentation of two discrete pure tones. One tone (CS+) was followed by a footshock US (unconditioned stimulus) whereas the other tone (CS–) was not. Importantly, for CS+ trials, if the rabbits stepped or hopped and produced wheel rotation of at least 2 degrees, the footshock US was not delivered. With this training, rabbits learned to make locomotor-conditioned responses (CR) on CS+ trials and withhold responses on CS– trials. The performance of the subjects was measured via the percentages of the conditioned responses to the CS+ and CS–.

Gabriel often referred to this procedure as “instrumental avoidance” (e.g., Poremba and Gabriel, 1999), however, the extent to which avoidance behavior was mediated by Pavlovian or instrumental learning is not clear (for a detailed discussion related to Gabriel’s studies, see Bradfield et al., 2013). For instance, rabbits may have learned a predictive relationship between the CS+ and footshock (CS-US), with the resulting CR being reflexively elicited by the CS. In contrast, they may have learned about an instrumental contingency, for example, that their own response prevented the occurrence of shock (response → no outcome). While these two accounts are indistinguishable during initial acquisition, two test criteria can identify which mechanism predominates: first, instrumental responses can be withheld during stimulus presentation and second, instrumental responses can be flexibly altered to obtain outcomes (e.g., performed in the opposite direction). Neither of these characteristics is true for Pavlovian CRs. As noted by Bradfield et al. (2013), Gabriel and colleague did not explicitly test these criteria, making it difficult to conclude which learning process governed behavior. Nevertheless, for the purpose of this paper, we take a neutral view on whether Gabriel’s studies captured Pavlovian or instrumental learning (this requires further experimentation). Instead, we emphasize that these studies provide insight into RSC contributions to aversive learning and memory in situations where a *discrete cue* controls performance of the CR.

### Overview of neural recordings: multi- and single-unit activity

Gabriel and colleagues recorded neural activity from the RSC (and related areas) of rabbits while they acquired and performed the discriminative conditioning task, thus providing a neural signal proxy at each stage of the experiments. In most cases, multi-unit activity (MUA) was recorded. MUA generally refers to electrical activity in the brain when neuronal firing is apparent, but the specific identity/number of neurons cannot be discerned. The resulting signal thus represents the *sum* of the firing activity of the local population of neurons (surrounding the electrodes), providing general information about the role of a region during aversive discriminative learning.

In one paper, Gabriel and colleagues reported analysis of single-unit activity (Kubota et al., 1996). Single-unit activity is the activity of isolated, putative single neurons while the animals perform/learn a task. In contrast to MUA, single-unit analysis involves isolating and tracking individual neurons, and therefore provides a high degree of resolution. For instance, single-unit analysis can provide insight into the excitatory and inhibitory activity of several individual neurons, but if this summed activity were equal, a multi-unit analysis would not detect changes in neuronal firing. Unless otherwise noted, the results described in the following sections will focus on MUA, considering most studies by Gabriel and colleagues reported neural activity in this way.

### Retrosplenial and avoidance learning findings: behavior and neural activity

In this section, we summarize the key findings of Gabriel and colleagues regarding RSC neural activity during discriminative avoidance behavior. Gabriel and colleagues generally Z-scored CS elicited firing against the pre-CS baseline data; thus, any increases in Z-score averaged neural activity indicates increased activity relative to baseline. Activity was assessed during behaviorally defined learning stages that included: pretraining, first conditioning, first significant discrimination, criterion, overtraining and reversal learning. In this review, we will collapse across phases of training, and summarize the findings based on three potential *functions* of the RSC during aversive conditioning. Specifically, (1) “non-affective” processing, (2) association of the CS with the aversive US, and (3) maintenance of the aversive memory. A detailed summary of neural activity during each training phase is presented in Tables 1, 2.

**Table 1:** Summary of multi-unit activity in the retrosplenial cortex during discriminative avoidance conditioning. Overtraining is not in the table because to the best of our knowledge Gabriel did not report layer specific neural recordings during this phase of the experiments. However, the RSC subdivisions (29c/d and 29b) show similar MUA patterns between criterion and overtraining phases. “+” represents the strength of neural activity, with maximal strength indicated as red “+++”. *Reversal laminar studies (Gabriel et al., 1980; Orona et al., 1982) did not specify in which area (29b vs. 29c/d) recording occurred. As such, we assume homogeneity across the subregions in this table, which may not be the case. #Pretraining represents the MUA levels used as the baseline for the subsequent phases. MUA during the pretraining phase represents changes between the CS period and the pre-CS period. ^Excitatory (non-discriminatory) TIA during reversal learning depends on if the animal experienced over-training. Higher TIA is observed in non-overtrained (non-OT) animals. The data is organized as such: Non-OT/OT (see Orona et al., 1982).

Excitatory TIA								
		Non-affective Processing	Associative Strength			Memory Maintenance		
		Pretraining#	First Exposure	First Discrimination	Criterion	First Reversal*	First Reversal Discrimination*	Reversal Criterion*
29c/d	Layer I	+	++	+++	+	++/+^	++/+^	++/+^
	Layer II/III	+	++	+++	+	++/+^	++/+^	++/+^
	Layer IV	+	++	++	+++	++/+^	++/+^	++/+^
	Layer V	+	++	+++	++	++/+^	+/Negligible^	+/Negligible^
	Layer VI	+	+++	+++	++	++/+^	+/Negligible^	+/Negligible^
29b	Layer I	Absent/Negligible				++/+^	++/+^	++/+^
	Layer II/III	Absent/Negligible				++/+^	++/+^	++/+^
	Layer IV	+	+++	+++	++	++/+^	++/+^	++/+^
	Layer V	Absent/Negligible				++/+^	+/Negligible^	+/Negligible^
	Layer VI	Absent/Negligible				++/+^	+/Negligible^	+/Negligible^

**Table 2:** Summary of multi-unit activity in the retrosplenial cortex during discriminative avoidance conditioning. Overtraining is not in the table because to the best of our knowledge Gabriel did not report layer specific neural recordings during this phase of the experiments. However, the RSC subdivisions (29c/d and 29b) show similar MUA patterns between criterion and overtraining phases. “+” represents the strength of neural activity, with maximal strength indicated as red “+++”. *Reversal laminar studies (Gabriel et al., 1980; Orona et al., 1982) did not specify in which area (29b vs. 29c/d) recording occurred. As such, we assume homogeneity across the subregions in this table, which may not be the case. # discriminative TIA in reversal learning is evident only in the short latency period (20–30ms) and those are represented here for non-OT animals. Blue indicates activity appropriate to original training and green indicates activity appropriate to the reversal.

Discriminative TIA								
		Non-affective Processing	Associative Strength			Memory Maintenance		
		Pretraining	First exposure	First discrimination	Criterion	First reversal*#	First reversal discrimination*#	Reversal criterion*#
29c/d	Layer I	Absent	++	+++	+	+++	+	+
	Layer II/III	Absent	++	+++	+	+++	+	+
	Layer IV	Absent	++	++	+++	+++	+	+
	Layer V	Absent	Monotonically increasing		+++	+++	+++	+
	Layer VI	Absent	Monotonically increasing		+++	+++	+++	+
								
29b	Layer I	Absent/Negligible				+++	+	+
	Layer II/III	Absent/Negligible				+++	+	+
	Layer IV	Absent/Negligible				+++	+	+
	Layer V	Absent/Negligible				+++	+++	+
	Layer VI	Absent	Monotonically increasing		+++	+++	+++	+

In general, RSC MUA during all the training stages often exhibited a common triphasic waveform pattern at the onset of the CSs (e.g., Gabriel et al., 1987). Specifically, the MUA to a 500 millisecond (ms) CS displayed an initial excitation (peaked 20 ms after CS onset) followed by an inhibitory pause (peaked 50–70 ms after CS onset) and a final excitation (beginning 90 ms after CS onset and lasting to the end of the CS; Gabriel et al., 1987). The amplitude of these peaks and troughs were dynamic, changing across phases as the discrimination was acquired and expressed (Gabriel et al., 1987). This triphasic firing pattern was not observed in the single-unit analysis, suggesting that the triphasic pattern is a consequence of the sum of the activity of the local population, and not an intrinsic property of the single-units (Kubota et al., 1996). What could contribute to this pattern of firing? Although not speculated by Gabriel and colleagues, the difference between the first and second excitation could represent distinct populations of neurons encoding different features of discriminative avoidance learning.

#### “Non-affective” processing

Most discriminative avoidance studies by Gabriel and colleagues included two pretraining sessions – the first session consisted of presenting two novel auditory cues on their own (i.e., no shocks); and in the subsequent second session, both auditory cues and shocks were presented but in an explicitly unpaired manner. Typically, there were no differences in behavioral responding (i.e., stepping causing wheel rotation) to either of the cues during the pretraining stages (Gabriel et al., 1989), and likewise RSC MUA did not differ between the two auditory cues.

Interestingly, there was often cue-evoked MUA to *both* auditory cues during pretraining. This increased firing activity could represent some form of non-affective coding by the RSC, perhaps akin to sensory processing of the auditory stimulus or attention/arousal encoding. In support of attentional/arousal encoding, Talk et al. (2004) exposed rabbits to 120 presentations of an auditory cue alone for three consecutive days (thus, a total of 360 presentations). During the first 30 cue alone presentations, the cue evoked a strong excitatory response. However, by the last 30 cue alone presentations, the cue-evoked activity was reduced and perhaps even slightly inhibitory. Thus, RSC neural activity changed with experience with the cue alone. However, because MUA represents the *sum* of firing activity of the local population of neurons, future work involving single-unit recordings will further elucidate if sensory coding of relatively neutral cues is present in the RSC.

#### Associative learning

**Figure 2:**
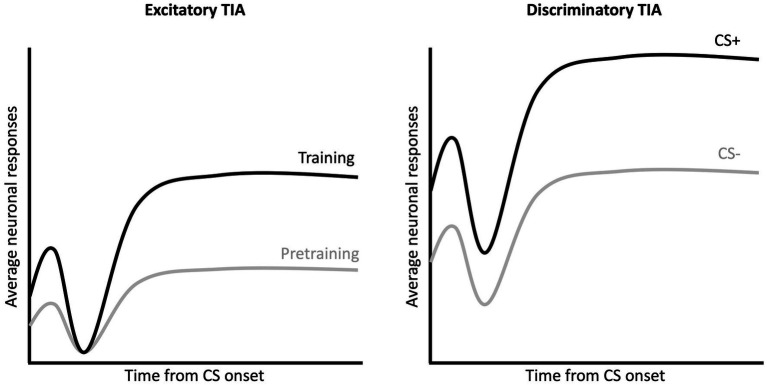
Excitatory and discriminatory training induced activity during CS presentation. Multi-unit activity in RSC exhibits a triphasic waveform in response to the auditory CSs, with an initial excitation (peak 20 ms after CS onset), an inhibition (peak 50–70 ms after CS onset) and a second excitation (90 ms to end of CS). Excitatory TIA **(left)** refers to the increase in MUA activity (for both CS+ and CS–) following training as compared to the activity recording during the pretraining sessions. Discriminatory TIA **(right)** refers to the greater activity to the CS+ as compared to the CS–.

Following pretraining, subjects received daily sessions in which one tone was followed by a footshock US (CS+) and one was not (CS–). Gabriel and colleagues described two distinct training-induced changes MUA that often first emerged during this training (Figure 2; Gabriel, 1990). Excitatory training induced activity (TIA) refers to increased *Z*-scored firing to *both* CS+ and CS– relative to the pretraining session (left panel of Figure 2). Discriminatory TIA refers to the greater increase in CS+ firing in contrast to the CS– (right panel of Figure 2). The two TIA patterns of firing may have related but distinct functions – with excitatory TIA a consequence of experience with both CSs and discriminative TIA a consequence of associative learning (i.e., prediction of shock vs. no shock). Gabriel and colleagues reported *maximal* excitatory and discriminative TIA in the RSC as the subjects acquired the discrimination and approached criterion performance. However, it is interesting to note that although RSC activity appears to encode the CS-US relationship, Gabriel et al. (1987) found that pre-training lesions of the RSC did *not* impact responding to the CS+ as rabbits reached criterion performance (lesions did increase responding to the CS–). Overall, these findings suggest that while the RSC encodes information about the CS-US relation during discriminative learning, the RSC may not be strictly necessary for performance of CRs to the CS+.

Kubota et al. (1996) analyzed and reported activity of single units during discriminative avoidance conditioning when the subjects had reached criterion. In area 29b/c, putative single-units had two responses, with neurons displaying both short- (10–90 ms after CS onset) and long-latency (90–690 ms after CS onset) discriminative firing to the CS+ vs. CS–. While only a small population of neurons were recorded (16 in area 29b and 37 in area 29c), about 50% of neurons exhibited short-latency and long-latency discrimination when analyzed separately. However, it is unclear how many of these neurons overlapped in terms of their discriminative properties. At criterion, RSC single units were also shown to exhibit a third, pre-avoidance, signal. Here there was differential firing on CS+ and CS- trials in the 2 s prior to onset of *conditioned response*, which may indicate a pre-motor signal in the RSC. All three neural response types were recorded only after the animals had reached criterion performance; hence it is unclear if the differential activity across CS+ and CS– is indeed due to associative learning. For instance, the differential activity may relate to the different sensory properties of the two CS. However, the observation in Kubota et al. (1996) of a slightly higher *proportion* of neurons displaying discriminative firing to CS+ than CS– is consistent with what was observed in discriminative TIA, and thus may be indicative of a neural code for the CS-US relationship. Therefore, activity in RSC the correlates with learning of discriminative avoidance with stronger TIA to CS+ than CS–.

#### Memory maintenance

In select studies, Gabriel and colleagues either conducted “overtraining” or reversal training following the criterion session (see Gabriel et al., 1973, 1983). “Overtraining” typically included 3 additional sessions of discriminative avoidance training. These sessions provided an opportunity to examine how the long-term maintenance of a well-learned discrimination is reflected in RSC neural activity. Unsurprisingly, both excitatory and discriminatory TIA were observed during overtraining, similar to what was seen during the criterion session, thus demonstrating a maintenance of the acquired associative structure in the MUA of the RSC (see control animals in Gabriel et al., 1987; Kang and Gabriel, 1998). Interestingly, Gabriel et al. (1987) observed that pre-training electrolytic lesions of the RSC reduced CRs to the CS+ during overtraining, suggesting that at this point in training the RSC is *necessary* for performance of the CR. Pre-training lesions have also been found to impair active avoidance in rats (Lukoyanov and Lukoyanova, 2006).

During reversal, the original CS- now predicted shock, and the original CS+ predicted no shock. During this phase, differences in discriminatory TIA emerged during the first peak of the triphasic pattern. In the deep the layers (V-VI) of the RSC, this discriminative TIA remained specific to original acquisition by maintaining the original activity (albeit with reduced amplitude) during reversal (Gabriel et al., 1980). However, in the superficial layers (I – IV) of the RSC, the discriminative TIA acquired during initial training showed changes that mirrored the behavior of the subjects during reversal. For instance, the sharp peak showed increased firing to the *old* CS+ during the first reversal session, but as the subjects reached criterion of reversal behavior, this peak of firing reversed and fired more to the *new* CS+ (Orona et al., 1982). Thus, there appears to be laminar differences in how reversal learning is encoded, with only the superficial layers of the RSC showing discriminative activity in line with the reversal task. This could represent a duality of learning signals in the RSC, with preservation of the original training signal in the deep layers while the new signal is represented in the superficial layer. Thus, there might be a long-term maintenance of the original memory trace in deep layers of RSC even in the presence of new learning.

### Network properties of discriminative avoidance

**Figure 3:**
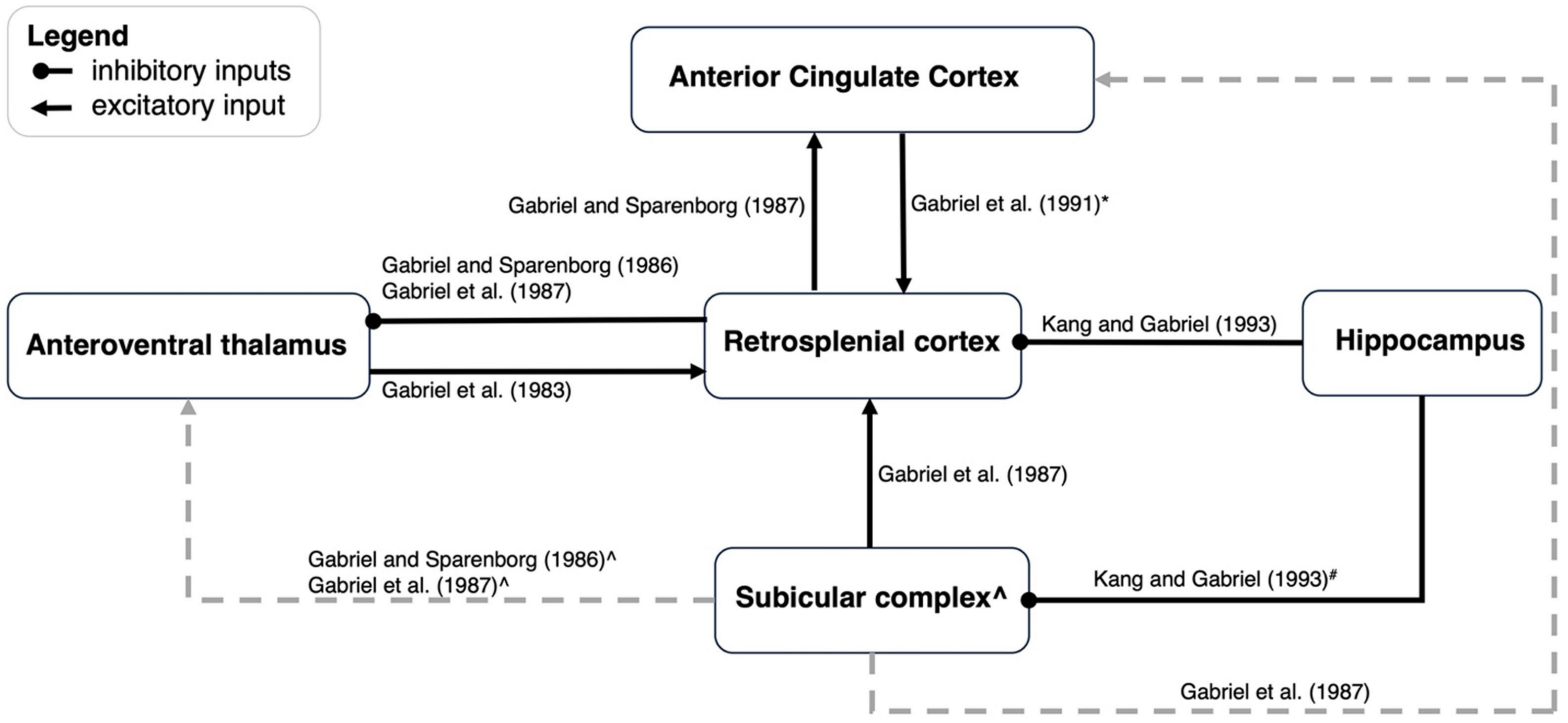
An updated schematic of network dynamics during discriminative avoidance behavior. This figure is an update of a schematic first presented by Kang and Gabriel (1998). Black arrows represent functional excitatory connections, and black lines ending with a dot indicate functional inhibitory connections. Grey lines refer to connections outside the scope of the present paper. *ACC to RSC projections appears to only contribute early in training. ^#^ Hippocampus to subicular complex connections have not been directly manipulated but are alluded to by Kang and Gabriel (1993). They suggested that lesioning of hippocampus could enhance excitation of AVN by way of the subicular → AVN projections, but also cause a limiting influence of RSC by way of the subicular → RSC --| AVN connection. The result is a modest enhancement of AVN activity. ^ Lesioning of the subicular complex leads to enhancement of AVN TIA, and as such, one would postulate that the subicular →AVN connection is inhibitory. *If* the subicular → AVN connection is indeed inhibitory, lesions of subicular complex should produce a stronger enhancement of AVN activity than lesions of the RSC alone. This is because subicular lesions would disinhibit AVN activity via the postulated inhibitory connection but *also* via a reduction of RSC activity, that will also disinhibit the AVN via the RSC --| AVN connection. However, Gabriel et al. (1987) observed greatest enhancement in AVN activity after RSC lesions, and less enhancement after subiculum lesions, thus suggesting an excitatory subicular input to the AVN.

Additional studies by Gabriel and colleagues examined the network properties of discriminative avoidance behavior by examining the impact of lesions of one region on neural activity in another region. Here, we have focused on the limbic regions, specifically structures that have direct connections with RSC: the anteroventral thalamus (AVN), subiculum, hippocampus, and the anterior cingulate cortex (ACC; see Figure 3 for a summary).

#### Effects of RSC lesions on neural activity

The influence of RSC lesions on the neural activity in other regions is indicative of the type of information that RSC may pass on to those regions. Gabriel and colleagues have investigated the influence of either RSC only (Gabriel and Sparenborg, 1986; Gabriel et al., 1987) or combined RSC and subiculum lesions (Kang and Gabriel, 1998) on AVN and ACC activity. Combined RSC and subiculum lesions resulted in increased discriminative TIA in the AVN from the first conditioning session, therefore indicating a limiting influence of RSC and subicular inputs onto AVN neurons (Kang and Gabriel, 1998). When only the RSC is lesioned, an increase in discriminative MUA to the CS+ is observed in the AVN during the timepoints when behavioral discrimination is maximal, thus the RSC may be limiting AVN specifically during situations when the subjects are performing optimally.

The ACC is reciprocally connected with the RSC, and also shows excitatory and discriminative TIA during discriminative avoidance (Gabriel et al., 1977, 1987). While lesions of the subicular complex impair TIA in the ACC (Gabriel et al., 1987), it may be that the RSC relays information from the subicular complex to the ACC, considering that the ACC receives strong input from the RSC, and minimal input from the subicular complex. Indeed, pre-training RSC lesions eliminated excitatory TIA and attenuated but did not fully eliminate discriminative TIA in the ACC (Gabriel and Sparenborg, 1986). RSC lesions tended to produce deficits on TIA in the early, but not late, stages of training suggesting that the RSC → ACC connections are critical for the first phases of behavioral acquisition.

#### Effects of other lesions on RSC activity

In other cases, Gabriel and colleagues examined how neural activity in the RSC was impacted by lesions of other regions. Subcortically, the AVN has bilateral projections with the RSC and both excitatory and discriminative TIA occurs in the RSC prior to its development in thalamic structures. Pre-training AVN lesions have no effect on behavioral acquisition of the CRs but impairs both excitatory and discriminative TIA within the RSC (Gabriel et al., 1983, 1989). These data provide evidence to support the idea that *neural responding* (i.e., TIA) in the RSC is not necessary for behavioral responding in an aversive discrimination paradigm.

Gabriel and colleagues have also examined how cortical lesions influence RSC neural activity during discriminative avoidance behavior. Lesions of the hippocampus impact MUA in the RSC, but interestingly, sub-regions of the RSC are impacted in different ways. In area 29c/d lesions of hippocampus enhanced discriminative TIA only during the first extinction session. In contrast, activity in the more ventral area of granular RSC (area 29b) undergoes complex changes following lesions of the hippocampus. This includes a deepening and lengthening of the inhibitory pause in the triphasic firing pattern as well as an enhancement of the theta-rhythmic excitatory peak that precedes the inhibitory pause (Kang and Gabriel, 1998). Such changes can be seen across several training stages, with the enhancement seen in lesioned animals during acquisition, extinction, and reacquisition, all of which are training stages in which there is a change in contingency. Thus, the hippocampus may exert a limiting influence on the RSC especially in area 29b and only during changes in contingency. The limiting influence could be the consequence of long-range inhibitory neurons from hippocampal CA1 (Jinno et al., 2007; Miyashita and Rockland, 2007; Yamawaki et al., 2019).

In contrast to the limiting influence of hippocampus on the RSC, the subiculum appears to function in concert with the RSC. Lesions of the subiculum lead to an attenuation of CS-elicited responses in RSC, which is more pronounced during early training relative to later training (Gabriel et al., 1987). Lastly, lesions of the ACC produce a reduction in RSC MUA activity early in training but do not reduce firing after the animals have reached criterion. This is consistent with the impact of ACC lesions on behavior; lesions of the ACC produce a mild retardation in the acquisition of discriminative avoidance (Gabriel et al., 1991).

## Part 2: RSC and Pavlovian fear conditioning

A relatively more recent literature has extended the prior work on discriminative avoidance, by examining RSC contributions to Pavlovian fear conditioning. These studies have primarily interrogated RSC contributions to aversive conditioning via “loss of function” studies (i.e., lesions; pharmacological, chemogenetic, and optogenetic inactivation), and in most (but not all) cases the conditioned response measured is freezing.

### Trace fear conditioning

Many of the discriminative avoidance studies described above used a *trace* conditioning procedure, in which there is a short interval following offset of the auditory cue and onset of the US. Relatively recent research has also demonstrated that the RSC is necessary for acquisition, retrieval, and extinction of Pavlovian trace fear conditioning. For instance, Kwapis et al. (2015) found that pre-training infusion of anisomycin into the RSC impaired trace fear conditioning. In addition, blocking NMDA receptor activity during a retrieval test session ~24 h after conditioning also impaired freezing to the trace CS (see also Kwapis et al., 2014). Extinction of trace fear conditioning increases phosphorylation of extracellular regulated kinase (pERK), and intra-RSC blockade of NMDA receptors impairs extinction learning (Kwapis et al., 2014). Finally, chemogenetic inhibition of the RSC also impairs retrieval of trace fear conditioning, when there is a lengthy retention interval between initial conditioning and final retrieval (Todd et al., 2016). These findings therefore implicate the RSC in the acquisition, extinction, and long-term retrieval of Pavlovian trace fear conditioning.

While the RSC is the one of the largest cortical structures in the rodent brain (Vann et al., 2009), recent research has started to parse specific functions of unique RSC “subregions” with respect to Pavlovian fear conditioning. For instance, Trask et al. (2021b) optogenetically silenced distinct “anterior” and “posterior” sections of the RSC during trace fear conditioning. They found that expression of fear to the trace CS was impaired during retrieval testing, but only for rats that had anterior silencing during conditioning. Thus, at least for encoding of trace fear, there appears to be a dissociation between anterior and posterior RSC. In a separate series of studies, Trask et al. (2021a) went on to examine the roles of anterior and posterior RSC in the *retrieval* of trace fear. In these studies, optogenetic inhibition of *either* anterior or posterior RSC impaired performance during retrieval testing. Taken together, this suggests that while the anterior RSC is necessary for both encoding and retrieval of trace fear, the posterior RSC is selectively involved in retrieval. This work is important as the anterior/posterior axis of the RSC receives quantitatively different amounts of inputs and may provide an insight into how these differential inputs contribute to aversive conditioning.

### Delay fear conditioning

In contrast to trace conditioning, delay conditioning involves contiguous presentation of the CS and US, such that CS offset is often coincident with US onset. Relative to RSC contributions to trace conditioning, the role of the RSC in delay fear conditioning appears specific to certain circumstances. For instance, permanent lesions of the RSC made prior to, or just after, conditioning typically do not disrupt the expression of conditioned fear to auditory or visual cues (Keene and Bucci, 2008, 2021; Todd et al., 2017; Jiang et al., 2018; Robinson et al., 2018). Selective manipulations of the RSC have produced the same results; blocking RSC protein synthesis prior to conditioning (Kwapis et al., 2015), and blocking NMDA receptors at the time of retrieval does not impact fear expression to a delay conditioned CS (Corcoran et al., 2011; Kwapis et al., 2014, 2015). Finally, optogenetic inhibition of either the anterior or posterior RSC also has no effect on the retrieval of delay fear conditioning (Trask et al., 2021a). Thus, across a range of manipulations, impairing RSC function appears to have no impact on delay fear conditioning.

There is, however, at least one exception to this rule. The studies of delay fear conditioning have all manipulated the RSC prior to, or shortly after, initial fear conditioning. Thus, conditioning has been acquired relatively “recently.” In contrast, several studies have now demonstrated that the RSC is necessary for the retrieval of delay fear conditioning that was acquired in the more distant past (i.e., a “remote memory”). In one study, rats underwent delay fear conditioning, and then were returned to their home cages for a 28-day retention interval (Todd et al., 2016). Different groups of rats then received either electrolytic, neurotoxic or control lesions. Following recovery, both lesion groups froze less to the tone CS during retrieval testing. Thus, damage to the RSC disrupts remotely acquired delay fear conditioning. Similar results have been found with delay fear conditioning to a visual stimulus (Jiang et al., 2018). These findings have been further extended by Fournier et al. (2021) who found that chemogenetic silencing of the RSC impairs the retrieval of remotely, but not recently, acquired delay fear conditioning. Further, Fournier et al. (2021) went on to show that silencing the RSC during *conditioning* did not impact retrieval of delay fear conditioning. Thus, taken together, the results of Fournier et al. (2021) suggest a selective role for the RSC in the retrieval of remotely acquired memories in delay fear conditioning.

Although the above studies suggest little role of the RSC in the encoding and retrieval of delay fear conditioning at *recent* time points, there is other evidence to suggest that delay conditioning may activate RSC neurons. For instance, Radwanska et al. (2010) paired whisker stimulation with mild-tail shock in mice. Following training, the number of c-fos positive cells was increased in the granular and dysgranular rostral RSC of mice that received CS-US pairings in comparison to several control groups. Of course, the training procedures (80 CS-US pairings) and stimuli (whisker stimulation, tail shock) in this experiment were very different from the fear conditioning studies noted above. But consistent with this study, Plakke et al. (2009) reported activation of the granular RSC (assessed via metabolic mapping) following delay eyeblink conditioning in rats. There appears to be converging evidence that, at least in some cases, the RSC is *activated* following delay fear conditioning.

## Part 3: putting it together: summary, discussion, and open questions

Here we have reviewed two literatures that have examined the role of the RSC in aversive learning and memory. In the first, electrophysiological recording and lesion studies probed the role of the RSC in discriminative avoidance learning. The second literature builds upon the first and has focused on the role of the RSC in Pavlovian fear conditioning. Considered together, the two literatures provide strong evidence that the RSC contributes to aversive learning and memory. Here we consider if there are general themes/principles that can describe the functional contribution of the RSC across both discriminative avoidance learning and Pavlovian fear conditioning.

As we have noted, we have specifically reviewed studies in *both* literatures in which a discrete cue controls conditioned behavior. Is it possible the RSC represents the sensory properties of these conditioned auditory and/or visual cues? One interesting observation is that in almost all of Gabriel’s work there was increased MUA in the RSC during the pretraining sessions, possibly indicative of sensory processing, which is consistent with the fact that the RSC receives input from both auditory and visual cortex (van Groen and Wyss, 1992, 2003; Todd et al., 2016). Furthermore, the spatial cognition literature has provided ample evidence of visual processing in the RSC. For instance, there is a vast literature of work demonstrating that RSC lesions impair visually guided behaviors (Vann and Aggleton, 2002, 2004; Hindley et al., 2014) as well as head direction cell cue control (Clark et al., 2010). Additional work has demonstrated that single-units in the RSC are visually responsive (Powell et al., 2020) or are responsive to visual landmarks (Vedder et al., 2017; Fischer et al., 2020). Taken together, these findings suggest the RSC, at least to a degree, might encode the sensory features of cues during conditioning. Indeed, this idea is consistent with prior work in which lesions or inactivation of RSC impaired sensory preconditioning, which requires learning about seemingly non-affective auditory and/or visual cues (Robinson et al., 2011, 2014; Fournier et al. (2020)). However, future work is needed to fully understand this, and can perhaps focus on auditory processing (since auditory cues are typically used in conditioning paradigms), as well as multi-modal processing, especially in the context of avoidance and/or fear conditioning studies.

Both literatures we have reviewed involve learning about a predictive relationship between a cue and an aversive outcome. Does the RSC specifically encode this cue – aversive outcome relation? The observation that training-induced MUA in the RSC increases over the course of avoidance to a CS+ is perhaps indicative of such coding. However, there is reason to question if this function of the RSC is *specific* to aversive conditioning. For example, a recent study found that putative single RSC neurons respond to cues that predict sucrose reinforcement, indicating that neural responses in the RSC are not specific to aversive outcomes (Yoshida et al., 2021). This is consistent with early studies by Gabriel and colleagues, who observed discriminative MUA in the RSC during a discriminative approach task (Freeman et al., 1996; Smith et al., 2002). In this procedure, water-restricted rabbits learned to extend their head to contact a waterspout during a CS+ but not a CS-. Considering the avoidance and approach tasks differed in valence of the outcome, and the response required, it seems unlikely that the RSC specifically encoded either the specific outcome or the specific response. Instead, neurons in the RSC might be encoding the “behavioral significance” of these cues, as they signal reinforcement and the need for a response, generally (see Smith et al., 2018 for a review). These findings make it clear that neurons in the RSC respond to cues that predict both positive and negative outcomes. It is currently unknown, however, if distinct populations of RSC neurons respond to cues that predict aversive or appetitive outcomes, or if RSC neurons encode the prediction of both outcomes.

In the Pavlovian fear conditioning literature, one consistent finding is that the RSC has a protracted role in memory retrieval. Damage or inactivation of the RSC, weeks after initial conditioning, has been found to impair conditioned responding across a range of conditions (see Todd et al., 2019 for a review). This finding is seemingly consistent with the discriminative avoidance literature. Here, CS-evoked RSC neuronal activity is maximal at late phases of training, and lesions of the RSC also tend to impact behavior at the late stages of training. In fact, Gabriel described the RSC as a posterior *primacy* or *reference system*, in which the RSC is critical for long-term memory storage of the original CS-US association (Gabriel, 1990; Freeman et al., 1996). However, because the avoidance studies consisted of training sessions over the course of several days, training itself (e.g., CS-US trials) covaried with the passage of time. Interestingly, Freeman and Gabriel (1999) examined how a retention interval (in the absence of continued training) impacted neural activity in the RSC. In this study, RSC activity was recorded in two groups of rabbits, each of which received three training sessions. The first session was pretraining, and the second session involved discriminative avoidance conditioning. For one group of rabbits, the third session occurred immediately after the second session (i.e., a 0-h retention interval). However, for a second group of rabbits, the third session occurred 48 h after the second session. In this study, discriminative MUA was observed during the third session for the 48-h group, but not the 0-h group. Although the absolute time intervals in the avoidance literature and the Pavlovian fear literature are quite different, the overall picture suggests a role for the RSC following a retention interval between initial conditioning and later retrieval (for a discussion see Todd et al., 2019). The notion that the RSC may have an important role in long-term memory storage is consistent with findings from other paradigms, such as contextual fear conditioning, in which the RSC is necessary for the retrieval of both recent and remotely acquired memories (Corcoran et al., 2011).

This review has focused on conditioned behavior controlled by discrete cues, such as tones. Therefore, we have not extensively reviewed RSC contributions to other aspects of learning and memory, such as contextual learning and spatial navigation. However, all learning and memory takes place against a set of background or “contextual” cues. Thus, in situations where learning and memory for a discrete cue is assessed, contextual cues may play an important role. Indeed, this notion was appreciated by Gabriel and colleagues, in their theoretical conceptualization of training-induced neural activity during discriminative avoidance conditioning. For instance, Freeman et al. (1996) described the training-induced neural activity as representing “a neural code of the spatio-temporal *context* that defines a particular learning situation” (pg. 1547 emphasis added). They went on to note that this idea proposes two conditions that must be met for a specific neural code to be activated: “(1) a particular spatial circumstance (the rabbits must be in a specific training apparatus), and (2) a particular temporal circumstance (a specific stage of behavioral learning, e.g., initial, intermediate, asymptotic, overlearned, etc.)” (pg. 1547). Thus, RSC involvement in discriminative avoidance learning may be intimately tied to contextual processing. This may also be true of the RSC’s role in Pavlovian learning. For instance, Todd et al. (2019) suggested that the RSC might integrate information about specific cues, outcomes, and the *contexts* in which they occur.

In summary, we have reviewed two literatures that demonstrate a role for the RSC in aversive conditioning. We have discussed several “themes” that are relevant to both avoidance learning and Pavlovian fear conditioning, while also noting several remaining questions. One promising area of research will be to determine RSC contributions to Pavlovian fear conditioning within a larger *network*. As reviewed above, such an analysis has been examined in the discriminative avoidance literature, however, little is known about how the RSC “fits” within a larger network supporting Pavlovian fear conditioning.

## Author contributions

HC: Writing – review & editing, Writing – original draft. DF: Writing – review & editing, Writing – original draft. TT: Writing – review & editing, Writing – original draft.
